# From Inflammation to Current and Alternative Therapies Involved in Wound Healing

**DOI:** 10.1155/2017/3406215

**Published:** 2017-07-25

**Authors:** Mariana Barreto Serra, Wermerson Assunção Barroso, Neemias Neves da Silva, Selma do Nascimento Silva, Antonio Carlos Romão Borges, Iracelle Carvalho Abreu, Marilene Oliveira da Rocha Borges

**Affiliations:** ^1^Physiological Sciences Department, Federal University of Maranhão, São Luís, MA, Brazil; ^2^School of Medicine, Emergency Medicine Department, University of São Paulo, São Paulo, SP, Brazil

## Abstract

Wound healing is a complex event that develops in three overlapping phases: inflammatory, proliferative, and remodeling. These phases are distinct in function and histological characteristics. However, they depend on the interaction of cytokines, growth factors, chemokines, and chemical mediators from cells to perform regulatory events. In this article, we will review the pathway in the skin healing cascade, relating the major chemical inflammatory mediators, cellular and molecular, as well as demonstrating the local and systemic factors that interfere in healing and disorders associated with tissue repair deficiency. Finally, we will discuss the current therapeutic interventions in the wounds treatment, and the alternative therapies used as promising results in the development of new products with healing potential.

## 1. Introduction

The immune system is composed of an organs network and cells and molecules that maintain the body's homeostasis. Factors that compromise the functionality of the immune system can make simple infections spread becoming fatal [1].

The major innate immunity cells that reach the site of injury are neutrophils and macrophages. These cells exert phagocytic activity, releasing highly destructive substances like enzymes that digest proteins, generating reactive chemicals products. When these cells fail to control infection, lymphocytes are activated and incorporate the adaptation and memory functions, allowing the immune system to elaborate increasingly specific responses [2].

The first defense of the organism to tissue damage is the inflammatory response, a complex biological process involving vascular and cellular components, and a diversity of soluble substances, which presents as characteristic clinical signs: flushing, heat, edema, pain, and functional impairment [3]. The purpose of this process is to remove the inducing stimulus from the response and initiate local tissue recovery. During inflammation, several biochemical systems are activated, such as complement and coagulation cascades, aiding in the establishment, evolution, and resolution of the process. In addition, soluble substances of short half-life are released, develop their action, and then are degraded. In general, successful removal of triggering stimulus leads to the end of acute response and tissue repair [4].

## 2. Inflammation and Tissue Repair

**Figure 1:**
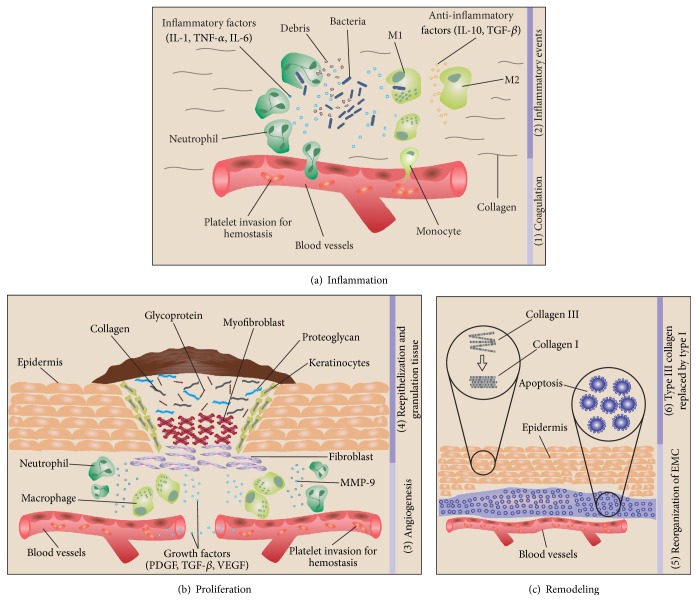
Progression and overlap of the phases involved in the physiological wound healing process: (a) inflammation begins with (1) coagulation, platelet aggregation, and fibrin clot formation; (2) then inflammatory events occur through neutrophils and macrophages infiltration and phagocytosis of debris, apoptotic cells, and pathogens; anti-inflammatory events occur through inhibition of destructive inflammatory process and proliferation promotion. (b) In the proliferation occurs (3) angiogenesis; (4) reepithelization (epithelial cell mitosis and fibroblasts transformation into myofibroblasts), and granulation tissue formation (EMC composed of collagen, glycoprotein, proteoglycan, fibroblasts, and keratinocytes, under modulation of MMP-9). (c) Remodeling is marked by the (5) EMC reorganization: cells apoptosis and angiogenesis regression; and (6) type III collagen replaced by type I.

Wound healing is a complex event that develops in three phases: inflammatory, proliferative, and remodeling (Figure 1). These phases are distinct in function and histological characteristics. However, they depend on interaction of cytokines, growth factors, chemokines, and chemical mediators from cells to perform regulatory events [5, 6].

The acute inflammatory response has an integral role in tissue healing, being fundamental for the homeostasis reestablishment [3]. Immediately after injury, vasoconstriction occurs with the substances release, such as serotonin, thromboxane A2, and prostacyclin by cell membranes, in order to prevent blood leakage. The exposed collagen signals the activation of coagulation cascade and in a coordinated way the platelets adhere to damaged blood vessels, initiating hemostasis, with the buffer formation composed of fibrin and thrombin. This buffer will have main functions, such as to prevent the cellular elements loss; to serve as physical barrier to microorganisms' entry; and to act as provisional matrix, cytokines' deposit and growth factors that will be fundamental for maintenance of other healing phases [4].

The inflammatory response begins with vasodilation, stimulated by soluble factors release such as nitric oxide, bradykinin, histamine, and E and I series prostaglandins. The increase in vascular permeability with consequent fluid loss leads to slow blood flow, allowing leukocytes, mainly neutrophils, to interact with endothelium in an events sequence involving margination (free leukocytes capture in the vascular lumen); rolling (weak interaction and activation); adhesion (firm interaction); transmigration (leukocyte passage through endothelial cells), and, finally, the targeting of leukocytes to focus of lesion under influence of several inflammatory mediators with chemotactic activity and alterations of vascular endothelial membrane proteins [7].

To achieve extravascular space towards the injured tissue, leukocytes depend on adhesion molecules expression such as selectins, integrins, and adhesion molecules of the immunoglobulin family [intercellular adhesion molecule-1 (ICAM-1) and vascular cell adhesion molecule-1 (VCAM-1)] [8]. Initially, the selectin mediates the capture and recruitment of leukocytes along the endothelial cells followed by the actions of ICAM-1 and VCAM-1 molecules to reduce leukocyte rolling velocity and allow its strong adhesion to endothelium [9].

In the first few hours after injury, neutrophils are recruited and mediate tissue damage through the release of proteases, cytokines, and other factors contained in cytoplasmic granules [10]. These cells generate reactive oxygen species (ROS) and produce antimicrobial proteases (cathepsins, defensins, lactoferrin, and lysozyme) with the function of destroying potentially pathogenic microorganisms. In addition, they release enzymes such as collagenases and elastases that aid devitalized tissues digestion, essential for tissue renewal in following repair phases [11].

Neutrophils also produce various types of membrane metalloproteinases (MMPs), having as main subtypes MMP-8, which cleaves fibrillar collagen, and MMP-2/MMP-9, which cleave collagen IV (among other substrates), both involved in the extracellular matrix degradation. The MMPs activity is inhibited by a molecules class called tissue inhibitors of metalloproteinases (TIMPs) produced by a cells variety on skin. If the proteases activity and their inhibitors are not strictly regulated the granulation tissue formation may be impaired [12]. Thus, uncontrolled neutrophil migration generates a cycle of recruitment and activation of these cells leading to excessive ROS and proteases production, causing undesired extracellular matrix degradation and additional tissue damage, which may progress to chronic inflammation with consequent defective collagen deposition, reduced tissue resistance, and late reepithelialization, limiting healing [13]. Moreover, they release cytokines such as tumor necrosis factor alpha (TNF-*α*), interleukin- (IL-) 1*β* (IL-1*β*), and IL-6, which amplify the inflammatory response by activating more neutrophils and cells such as macrophages, and which, although essential for the repair cells activation, can generate deleterious effects when exacerbated release occurs [5].

In intact skin, macrophages are the most abundant cell types performing sentinel and homeostatic function. In skin lesion case, the monocytes migrate from vascular circulation to wound. Both infiltrating and resident macrophages on skin are activated by local signals and develop into several subpopulations defined by their different functional phenotypes [14]. Pathogen-associated molecular patterns (PAMPs) expressed by microbes and danger-associated molecular patterns (DAMPs) produced during cell stress activate macrophages in classic form, type M1, that act as host defense, performing phagocytosis, cleaning dead cells and debris, and producing proinflammatory mediators such as IL-1, IL-6, IL-12, TNF-*α*, and inducible nitric oxide synthase (iNOS), as well as chemokines to recruit additional leukocytes [15]. In contrast, cytokines, such as IL-4 and IL-13, lead to macrophages formation of the M2 subset, which regulate inflammation by expressing mediators as IL-1 receptor antagonist (IL-1R), IL-1 type II receptor, transforming growth factor-*β* (TGF-*β*), vasopressin endothelial growth factor (VEGF), and insulin-like growth factor (IGF-1), promoting fibroblasts proliferation, extracellular matrix synthesis, and angiogenesis [16, 17].

As the inflammation resolution occurs, the involved cells enter into apoptosis initiating the proliferative phase, which consists of four fundamental stages: reepithelization, angiogenesis, granulation tissue formation, and collagen deposition. This phase is characterized by intense cellular activity aiming to repair the connective tissue and to form granulation tissue and epithelium [18].

During reepithelialization, the keratinocytes migration from wound and epithelial attachments stimulated by growth factors release are mainly responsible for mitoses increase and epithelial hyperplasia [19]. Fibroblasts migrate to provisional matrix to degrade it, proliferating and producing MMPs. They also produce collagen, proteoglycans, hyaluronic acid, glycosaminoglycans, and fibronectin to form granulation tissue, which fills the wound space and provides support for cell adhesion, migration, growth, and differentiation during wound repair [20].

Angiogenesis is essential for nutrition and oxygenation of new tissue being formed. The formation of new blood vessels is initiated by growth factors, such as VEGF, platelet-derived growth factor (PDGF), and basic fibroblast growth factor (bFGF). After secreting proteolytic enzymes to dissolve the basal lamina, endothelial cells escape from existing blood vessels, proliferate and migrate to the source of angiogenic stimulus, and providing oxygen for maintenance of cellular functions [21].

Collagen production starts from time of granulation tissue formation through production, deposition, digestion, and reorganization steps. Initially, the collagen fibers are deposited in disorganized form, following a fibronectin model. Subsequently, in the attempt to organize these, they undergo digestion through enzymes of action produced by neutrophils, macrophages, and fibroblasts [18]. Next, new fibers will be produced and deposited in more organized way, following the adjacent connective tissue, initiating the remodeling phase [22].

The remodeling phase occurs most expressively at the end of granulation tissue formation step. Tissue development, increased mechanical stress and cytokine expression, such as TGF-*β*, stimulate fibroblasts to differentiate into myofibroblasts, which express a smooth muscle actin with contractile function, favoring the locomotion of these cells from edges to lesion center for wound contraction [23]. At this stage, the collagen III produced rapidly in the extracellular matrix is replaced by collagen I, which has a higher tensile strength, but takes more time to deposit [22]. The new collagen will be composed of larger fibers with greater fibrils number and with significant amount of cross-links between them, characterizing an increase in fiber diameter and tensile strength acquired by scar [24].

## 3. Regulatory Factors Involved in Inflammation and Healing

Wound healing is strongly regulated by a large number of cytokines and growth factors, acting as important mediators of differentiation, proliferation, and maintenance of important cells in repair process through various mechanisms [25].

There are currently 11 members of IL-1 family, of which IL-1*α* and IL-1*β* are the most described, differing in the way they are activated and function: IL-1*α* is translated into a biologically active protein and IL-1 *β* is translated as a propeptide that requires processing by caspase-1 enzyme in the inflammasome [7, 26]. IL-1*β* is a key interleukin of antimicrobial response by inflammatory response amplification; it stimulates leukocyte recruitment, the acute phase proteins release, and the increase of blood vessels permeability, as well as stimulating Cox II expression and, as a consequence, the prostanoids formation and release [27–29].

Prostanoids have a central role in inflammation, blood coagulation, angiogenesis, wound healing, vascular tone, and immune responses, among others [30, 31], and the suppression of their actions has been one of the main therapeutic targets for anti-inflammatory drugs development [32].

TNF-*α* is pleiotropic cytokine produced by a cell types variety, including keratinocytes, macrophages, and mast cells. It acts on several stages of leukocyte recruitment mechanism, mainly neutrophils, inducing molecular adhesion regulation, chemokine production, and metalloproteinases matrix, as well as tissue inhibitors of metalloproteinases. TNF-*α* may act in a beneficial or deleterious way in wound healing and its elevation leads to decrease in granulation tissue production while its reduction promotes a better collagen fibers arrangement. Another function of this factor is to suppress TGF-*β* in the stimulation of extracellular matrix (ECM) production, but on the other hand it indirectly acts on reepithelialization by inducing keratinocyte growth factor production, together with IL-1 [25, 33].

The keratinocyte growth factor (KGF) or fibroblast growth factor-7 (FGF-7) is an important member of FGF family involved in wound repair. The injured epithelium repair also depends on the mitogenic potency of KGF, which is produced by dermal fibroblasts and acts by stimulating keratinocytes proliferation through receptors present in these cells [33, 34]. KGF gene expression by dermal fibroblasts is increased after cytokine signaling, where some of these make part in IL-1 family [35].

IL-8, which also acts as a chemokine (CXCL8), is mainly produced by monocytes/macrophages and in smaller amounts by fibroblasts, endothelial cells, keratinocytes, melanocytes, hepatocytes, and chondrocytes. Their stimulation is usually IL-1, TNF-*α*, and IFN-*γ* (interferon-gamma) [10]. The main action of IL-8 is great migratory motivation for immune system cells, mainly neutrophils, also determining an increase in the expression of adhesion molecules by endothelial cells [11].

Migration and cell proliferation are growth factor dependent mechanisms. TGF-*β* inhibits matrix proteins degradation, decreasing MMPs synthesis and increasing TIMPs production [36]. Low concentrations or suppression of TGF-*β* exert a potentially negative influence, indicating some disturbance in the repair process [37].

The VEGF family proteins act as angiogenesis regulators during cellular development [38]. In response to hypoxia caused by injury, VEGF is released by macrophages, fibroblasts, and epithelial cells, resulting in increased nitric oxide and endothelial progenitor cells mobilization [21]. Angiogenesis, formation of new blood vessels from preexisting vessels, is an important phenomenon for the cicatrization proliferative phase by temporarily increasing the vessels number at lesion site, favoring oxygen and nutrients flow, toxin removal, cell migration, and signal transduction [39], contributing fundamentally to tissues growth and regeneration. However, when uncontrolled, it also contributes to pathologies progression such as arthritis, psoriasis, and cancer, being regulated by numerous pro- and antiangiogenic factors which are in equilibrium under normal conditions [39–41].

However, situations such as wound healing, growth-related hypoxia, and inflammation cause imbalance, inducing several proangiogenic factors activation, such as cytokines, lipid mediators, and growth factors. Skin regeneration during the wound healing process and bone regeneration are examples that an increase in angiogenesis level can accelerate and improve the outcome while avoiding necrosis [42].

One of the most important proangiogenic mediators is vascular endothelial growth factor (VEGF) by stimulating the endothelial cell functions necessary for new blood vessels formation, as well as for tissue proliferation, migration, differentiation, and survival, contributing to both angiogenesis and influencing wound repair and closure, and granulation tissue formation [43].

VEGF is produced in response to lesions by a cells variety, including keratinocytes, macrophages, and fibroblasts, developing various roles in the healing process. Acutely, they increase vascular permeability, adhesion cells expression, and selectins, recruiting inflammatory cells such as macrophages and mast cells, important in several stages of healing [44–46]. In the proliferative phase, it was verified that VEGF regulates several aspects, including epidermal repair and dermis barriers, acting directly on keratinocytes and macrophages which also express receptors (VEGFRs), whose cellular activities are also favored by oxygen and nutrients carried by new blood vessels [47, 48].

It is believed that VEGF levels present in wound can have a healing impact. Insufficient vascularization has been associated with abnormally low levels of active VEGF protein in individuals with wound closure difficulties, reduction in granulation tissue reepithelialization, and formation commonly in diabetic patients [49, 50]. In addition, drugs used to block VEGF activity, as in cancer treatment cases, represent a significant risk for tissue repair process [51]. After topical treatment with recombinant VEGF or via viral vector or liposome mediated gene transfer, there was acceleration in wound closure, granulation tissue increasing, and improving the resistance to wound rupture, influencing deposited collagen production or arrangement [50, 52–54].

One critical feature of remodeling phase is ECM remodeling to an architecture that approaches normal tissue [55, 56]. The known regression signals include soluble and ECM-derived antiangiogenic mediators which lead to specific intracellular signaling pathways that result in cellular and microenvironmental changes associated with vessel regression [57, 58].

Once the endothelial cells (ECs) are primed by hypoxia and activated by VEGF during the proliferative phase, there are probably several redundant intracellular negative feedback mechanisms protecting ECs from VEGF overstimulation during the postproliferative and remodeling phases of healing which help to guide them into regression [59, 60]. It appears that the postproliferative wound not only becomes more resistant to proangiogenic stimuli by negative feedback mechanisms, but also generates active antiangiogenic signals [61].

Interestingly, the proangiogenic mediator VEGF may be one of factors responsible for vessel regression initiation in postproliferative phase. Studies have found that ECs activation by VEGF simultaneously marks these cells for death by induction of death receptor Fas, also known as CD95, which initiates apoptotic signaling pathways [59, 60], making them less resistant to death by apoptosis-promoting signals.

Finally, different fibroblast subpopulations may play a role in determining the fibroblast's pro- or antiangiogenic functions. Fibroblasts derived from papillary dermis and cocultured with ECs are angiopermissive, stimulating robust vessel growth, whereas reticular fibroblasts from deeper tissue are angiorestrictive, presumably because of nonsoluble factors such as secreted ECM composition. At the wound resolution phase onset, fibroblasts may switch to an antiangiogenic phenotype due to contact inhibition and normalizing oxygen levels to regulate ECM remodeling, indirectly mediating vessel regression [62]. Besides the soluble and matricellular factors, an essential class of antiangiogenic molecules are those derived from ECM components, generated when specific matrix proteases cleave large ECM proteins into bioactive peptides [63].

Several antiangiogenesis factors act in different signaling pathways to regulate endothelial cell proliferation, migration, and survival as well as help limit excessive angiogenesis during wound healing, inflammation, and disease processes:

1. Inhibits the migration and/or proliferation of endothelial cells: chondromodulin-I (ChM-I) [64]; pigment epithelium-derived factor (PEDF) [65]; vasostatin [66]; antiangiogenic matricryptins derived of type IV collagen, including arresten, canstatin, and tumstatin [67–69]; tissue inhibitor of matrix metalloproteinase (TIMP) inhibits extracellular matrix degradation and remodeling which is necessary for efficient endothelial cells migration and proliferation [70, 71]; endostatin, was found to inhibit the migration, but not the proliferation, of endothelial cells in vitro and disrupt tumor vascularization and growth in mice [72]; thrombospondin-1 inhibits angiogenesis through direct effects on endothelial migration and survival through indirect effects on growth factor mobilization [73]
2. Induces apoptotic death of endothelial cells: interleukin 12 [74]; plasminogen Kringle 5 [75]; interferons (*α*, *β*, and *γ*) [76]; in addition to these, PEDF, involved in apoptosis stimulation in endothelial cells through NF-*κ*B, PPAR*γ*, and p53 mediated processes while inhibiting their proliferation and migration [77–79]
3. Reduction expression of angiogenesis activators: interferons (*α*, *β*, and *γ*) decreased expression of angiogenesis activators such as bFGF [80]; a soluble form of VEGFR1 (sVEGFR1) with high affinity for VEGF-A has been shown to inhibit angiogenesis through the regulation of VEGFR2 activation and inhibition of downstream mitogenic activities [81]; endostatin blocks action of VEGF [82] whereas interleukin-10 downregulates synthesis of VEGF and matrix metalloproteinase 9 (MMP-9) [83].

Angiostatin, derived from plasminogen, able to suppress proliferation and migration, induces endothelial cells apoptosis [84, 85] and additionally downregulates VEGF expression [86–88]. Two members of thrombospondin family, TSP-1 and TSP-2, are relatively well-studied potent antiangiogenic factors. These molecules had been found to inhibit angiogenesis by downregulating EC proliferation and migration, inhibiting VEGF signaling and initiating apoptosis [89]. Whereas TSP-1 is produced during the early phases of healing and likely functions to attenuate VEGF-mediated proangiogenic signals, TSP-2 is produced during the remodeling phase and is likely more involved in ECM remodeling-associated vessel regression [90].

IL-10 is a regulatory cytokine produced by different cells, capable of inhibiting the activities of Th1 cells, natural killer cells, and M1 macrophages, but stimulates M2 macrophages to produce VEGF, aiding in increased angiogenesis [91, 92]. It can also inhibit the production of other proinflammatory cytokines, such as TNF-*α*, IL-1*β*, and IL-6 [91]. In addition to its potent anti-inflammatory effects, IL-10 has been shown to regulate fibrogenic cytokines, such as transforming growth factor-*β* (TGF-*β*), as a part of its role in the regulation of tissue remodeling [93]. Preclinical and clinical studies have shown that rhIL-10-treated rat incisions healed with decreased inflammation, better scar histology, and better macroscopic scar appearance. RhIL-10-treated human incisions at low concentrations healed with better macroscopic scar appearance and less red scars [94].

## 4. MicroRNA and Wound Healing

MicroRNAs (miRs) are approximately 22 nucleotides (nt) not encoding RNAs that bind to the 3′-untranslated regions (3′-UTR) of target messenger RNA (mRNA) and result in posttranscriptional regulation of gene expression [95] and have been found to regulate a variety of cellular and physiological functions in heath and disease. The miRs expression deregulation has been shown to be associated with various diseases. During wound healing, microRNAs play versatile roles but their functions are not yet understood [96]. The ability to therapeutically manipulate the miRs expression through administration of inducers and/or inhibitors showed excitement about the therapeutic potential of miRs for nonhealing wounds [97].

MicroRNAs are present in all tissue types and regulate a wide variety of processes at the cellular level, including proliferation, differentiation, and apoptosis [98]. The miRs act as agonists and antagonists in the process of restoring skin barrier function. Changes in the specific miRs expression during different phases may be associated with abnormal wound healing [99].

Several pieces of evidence support that miRs regulate signals in wound healing phases. (1) In the inflammatory phase, macrophages are regulated by miR-146a and miR-155, which promote cytokines and growth factors production necessary for monocyte differentiation into macrophages [100, 101]. Toll-like receptor-4 (TLR-4)-mediated inflammation is regulated by miR-21 effects on programmed cell-death protein 4 (PDCD4) expression [102]. miR-146a, miR-155, and miR-21 are reported to be linked to wound healing processes [103, 104]. While miRs promote and induce inflammation, they also downregulate and terminate the phase [101]. (2) In the proliferation phase, new blood vessels begin to form to promptly provide the healing area with abundant oxygen and nutrients through angiogenesis/neovascularization [105]. Several studies have identified miRs in the regulation of various aspects of the angiogenic response to various pathophysiological stimuli. For example, miR-92a, miR-217, miR-221, and miR-222 inhibit angiogenic activity in endothelial cells (ECs), whereas miR-126, miR-130a, miR-210, and the miR-23–miR-27–miR-24 cluster promote proangiogenic activity [106–112]. Furthermore, keratinocytes migrate from the edge of the wound to the wound site and begin to proliferate and differentiate to restore skin integrity, a process that can be inhibited by several miRs, including miR-198, miR -203, and miR-483-3p [113–115]. (3) The remodeling phase begins when wound is closed [103]. miR-29a regulates dermal fibroblasts by their contractility control through TABL1 [116]. miR-192/215 increases E-cadherin expression by repressed translation of ZEB2 [117], while E-cadherin plays a role restoring the skin barrier integrity. The discovery of several miRs involved in the remodeling phase regulation still requires further investigation.

MicroRNA 26a (miR-26a) has been reported to participate in normal development, metabolic process, and wound response [118]. Furthermore, miR-26a also regulates the growth of endothelial cells during physiological and pathological angiogenesis by targeting BMP/SMAD1 signaling [119]. Also a role for miR-26a in regulation of diabetic wound healing progression was identified. miR-26a expression is induced in diabetic mice wounds and its neutralization promote wound closure through increased granulation tissue, induction of SMAD1 signaling in ECs, and enhanced angiogenesis. These findings indicate miR-26a therapeutic inhibition as promising treatment for diabetic subjects with impaired dermal wound healing [96].

Though miRs could be new potential therapeutic target for wound healing, it is still far from being a real application, and further studies are needed to identify the miRs involved in each wound healing phase.

## 5. Toll-Like Receptors in Wound Healing

Toll-like receptors (TLRs) are a group of pattern recognition receptors (PRRs) highly conserved that indicate the presence of several pathogen-associated molecular patterns (PAMPs) to cellular constituents of the immune system. After binding to different biochemical components of protozoa, bacteria, and viruses, TLRs via NF-*κ*B-dependent and interferon regulatory factor- (IRF-) dependent mechanisms trigger immune responses. Moreover, TLRs are also activated by endogenous ligands called damage-associated molecular patterns (DAMPs) that they are inaccessible to the immune system under physiological conditions or undergo changes in response to injury, leading to recognition by PRRs. Following tissue injury, these patterns are unmasked or released from damaged cells and subsequently trigger inflammation via TLRs and other PRRs. Consequently, TLRs can be considered as master safeguards of tissue structural integrity: activated by molecular indicators of infection or injury that play a key role in the initiation of wound repair [120].

TLR activation in wound healing appears to be mediated by two classes of ligands. (1) In organs such as the gut, skin, and liver that are in direct contact with microbial products, tissue lesions lead to a protective barriers breakdown and consequently activation of TLR by bacteria PAMPs. (2) In many organs, such as the liver, heart, and kidney, the tissue injury leads to DAMPs release from dead cells, resulting in TLRs activation. The endogenous TLR ligands release occurs predominantly after tissue damage, especially in situations where a significant portion of cells undergo necrosis, such as ischemia-reperfusion injury [121–123].

According to their biological actions, the TLRs were implicated in different phases of wound healing: TLRs activation modifies tissue injury in positive or negative way by recruiting inflammatory cells that release cytotoxic mediators or by activating cytoprotective signals, enhances fibrogenic responses in fibroblasts, and promotes regenerative responses [124, 125].

Several lines of evidence support that TLRs regulate signals in wound healing. (1) Topical application of TLR3 agonist poly-(I:C) for wound closure in mice promotes reepithelialization, granulation, and neovascularization. Remarkably, topical application of poly-(I:C) in patients with laser plastic surgery accelerates wound closure [126]. On the other hand, mice without TLR3 exhibit delayed wound healing parameters, such as neovascularization, granulation formation, and reepithelialization [127]. (2) Nucleic acids, released by damaged skin wound cells, stimulate TLR7 and TLR9 in infiltrated plasmacytoid dendritic cells, leading to the transient production of type I interferon (IFN). Pharmacologic inhibition of TLR7 or TLR9, or deficiency of MyD88 and TLR7, inhibits type I IFN production. The presence of dendritic cells and production of type I IFN are required for reepithelialization [128]. TLR9 knockout mice exhibit a general delay in wound healing. Furthermore, administration of the TLR9 CpG ODN agonist promotes influx of macrophages to the wound site and increases the production of vascular endothelial growth factor, accelerating neovascularization of the wound in mice [129] and wound closure in nonhuman primates [130]. (3) Excisional skin wounds in MyD88^−/−^ mice heal by slower rate than wounds in wild-type MyD88^+/+^, showing delayed contraction, diminished and delayed granulation tissue, and reduced density of fresh blood vessels [131]. (4) In vitro and in vivo data has showed that TLR4 becomes upregulated within the first 12–24 hours following injury and slowly decreases at 10 days and is mainly concentrated in epidermal keratinocytes. The same study evidenced significant deterioration of wound healing in TLR4 deficient mice at days 1–5, and no difference shown from wild-type at 10 days [132]. Another study also observed impairment in wound healing in TLR2 and TLR4 deficient mice on days 3 and 7 [133]. The TLR4 and TLR2 activation appears to have a beneficial effect on wound healing in the early stages following acute injury [134]. (5) The TLRs stimulation plays an important role in promoting normal wound healing, but that excessive TLR signaling contributes to maladaptive or hypertrophic wound healing and fibrosis [135].

Evidences suggest that TLRs have important roles in wound healing and modulate the innate immune response. Nevertheless, they differ in their expression pattern, signaling pathways, cellular localization, and physiological outcomes on wound healing. It will be important to identify the TLRs impact on healing and innate immune responses [135]. This will improve the therapeutic strategies for the treatment of wound healing.

## 6. Healing Disturbances

The factors that influence tissue repair can be classified as systemic or local [136]. By approaching factors that affect healing locally, it is important to note that oxygenation modulation is very important for repair of cell maintenance activities by stimulating cellular metabolism, especially energy production by means of adenosine triphosphate (ATP), and is critical for almost all wound healing processes, acting to prevent infections, increasing angiogenesis, keratinocyte differentiation, cell migration, and reepithelialization [137].

Due to vascular ruptures and the high oxygen consumption by metabolically active cells, the microenvironment at cicatrization beginning has a greater need for oxygenation. At this time, low oxygen flow (hypoxia) is temporarily important for healing, but if prolonged, as in some pathologies, it can make the wound chronic and difficult to heal [138]. Hypoxia can induce cytokines expression and production of growth factors released by macrophages, keratinocytes, and fibroblasts. Cytokines that are produced in response to hypoxia include PDGF, TGF-*β*, VEGF, TNF-*α*, and endothelin-1, which are promoters of cell proliferation, migration, chemotaxis, and angiogenesis [139].

A factor that may negatively affect wound healing is infections presence, which may result in inflammatory phase prolongation and increased production of reactive oxygen species (ROS) and proinflammatory cytokines, such as IL-1 and TNF-*α*, induced by both bacteria and endotoxins present on site. If infection does not resolve, the wound may become chronic with persistent inflammation. The bacteria presence in the lesion may also be associated with bacterial biofilm formation, which creates a resistance microenvironment to medications action, making healing even more difficult [140].

Systemic factors that may interfere with healing may be age-related. It has been observed that elderly healing is associated with modified inflammatory responses, such as cells late infiltration in wound area, chemokine production changes, phagocytes reduction, delayed reepithelialization, and impaired collagenization [141].

Sex also influences healing through hormonal regulation on a variety of genes associated with regeneration, matrix production, regeneration, epidermal function [142], and protease inhibition [143] and genes associated primarily with inflammation [144]. It has been found that topical 17*β*-estradiol enhances mRNA and procollagen type 1 protein expression significantly in aged human skin. Expressions of TGF-*β*1 and TGF-*β* receptor type II were also increased, and TNF-*β*1 neutralizing antibody inhibits 17*β*-estradiol induced procollagen synthesis in cultured fibroblasts. Topical estradiol also increased the keratinocytes proliferation and epidermal thickness in aged human skin, also observing the same effects in young skin [145]. In addition, the elderly estrogen deficiency is also associated with healing difficulty [146].

Stress is another factor that can critically influence healing, as it is associated with increased glucocorticoids (GCs) and reduced proinflammatory cytokines levels (IL-1*β*, IL-6, and TNF-*α*) in the wound. It also reduces the IL-1*α* and IL-8 expression, both chemoattractants required for the initial inflammatory phase [147]. In addition, GCs influence immune cells by suppressing differentiation and proliferation, regulating gene transcription, and reducing the expression of cell adhesion molecules [148]. Stress has been shown to reduce T cell proliferation and T cell dependent antibodies production [149], besides increasing the phagocytic abilities of cells, and the number of neutrophils in the wound area of mice subjected to stress restriction [150].

Diabetes also interferes negatively in the wound healing process and many factors have been shown to be involved in the poor wound healing ability of diabetic patients, including hyperglycemic environment, chronic inflammation, wound infection, vascular insufficiency, hypoxia, sensory neuropathy, and abnormal neuropeptide signaling [151–153].

It has been postulated that hyperglycemia can lead to a deleterious effect on wound healing through the formation of advanced glycation end-products (AGEs). These end-products are a heterogeneous compounds complex group that are formed when reducing sugar reactions in a nonenzymatic way with amino acids in proteins and other macromolecules. This occurs both exogenously (in food) and endogenously (in humans) with greater concentrations found in older adults [154, 155]. These end-products reduce the solubility of the extracellular matrix and perpetuate the inflammatory alterations observed in diabetes [156, 157]. The AGEs also stimulate the proinflammatory molecules release, such as TNF-*α* and MMPs, which limit wound closure. In addition, the AGE-RAGE (AGE receptor) interaction in fibroblasts may cause reduction of collagen deposition, further compromising the normal healing process [158].

An altered immune function may also contribute to poor wound healing in patients with diabetes. Studies suggest that a failure in removal of inflammatory cells, such as neutrophils, plays a role in the pathogenesis of nonhealing wounds. A deficit in the capability of macrophages to effectively remove neutrophils has been reported to be a critical component of the impaired healing seen in diabetes [159, 160]. Other studies have shown that the prolonged inflammatory phase is characterized by sustained expression and increased levels of proinflammatory cytokines, such as interleukin-1 (IL-1), interleukin-6 (IL-6), and tumor necrosis factor-*α* (TNF-*α*) in diabetics [161, 162].

Decreased peripheral blood flow and diminished local neovascularization are critical factors that contribute to the delayed or nonhealing wounds in diabetics. Endothelial progenitor cells (EPCs), a specialized subset of hematopoietic progenitor cells (HPC), are the key cellular effectors of ischemic neovascularization and play a central role in wound healing [163]. EPC are capable of inducing endothelial differentiation [164] and secretion of angiogenic growth factors and cytokines [165, 166], which are of paramount importance in neovascularization. The circulating number of EPC and wound level are decreased in diabetes, implying an abnormality in EPC mobilization. This deficiency in EPC mobilization is presumably due to impairment of endothelial nitric oxide synthase (eNOS-NO) cascade in bone marrow (BM) [163].

Abnormal expression of growth factors has been observed in diabetics. Insulin-like growth factor I (IGF-1; a growth factor similar to insulin) is a cytokine that participates in the cellular granulation process during wound healing. The IGF-1 anabolic effects include stimulation of DNA synthesis, cell proliferation, protein synthesis, and glucose transport. During healing, its expression is increased. However, diabetic patients show overall decreased levels of IGF-1 expression [167].

Disturbed physiologic functions of epidermal keratinocytes also have been found to play an important role in the poor healing ability of diabetic wounds [168]. Factors involving keratinocytes that may contribute to the dysfunctional wound healing process in diabetes include impaired keratinocyte migration and proliferation, gap junction abnormalities, chronic inflammation, chronic infections, reduced angiogenesis, oxidative stress, and abnormal expression of MMPs [153, 169–171].

Some drugs that interfere with clot formation or platelet function, or inflammatory responses and cell proliferation, have the ability to affect wound healing. Systemic glucocorticoids, frequently used, can inhibit tissue repair by suppressing immune cells, complicating cell signaling that compromises the other healing stages, such as fibroblast proliferation and collagen synthesis. Systemic steroids cause scarring, incomplete granulation tissue, and wound contraction reduction [172].

Nonsteroidal anti-inflammatory drugs (NSAIDs), such as ibuprofen, are widely used for inflammation and pain treatment and rheumatoid arthritis. Low-dose aspirin, because of its antiplatelet function, is commonly used as a preventive therapy for cardiovascular disease, but not as an anti-inflammatory [173]. However, it is important to be cautious with the use of these drugs during healing, as they may affect the inflammatory phase, making hemostasis and clot formation difficult at the process beginning [24].

Chemotherapeutic drugs also negatively interfere in wound healing, since they are associated with delayed cell migration, extracellular matrix formation impairment, collagen production, fibroblast proliferation, and inhibition of wound contraction [174]. Other factors such as alcoholism [175], smoke [176], and precarious nutrition [177], as well as obesity [178], vascular diseases [179], and metabolic syndrome [180] are also associated with healing damage.

Disorders of wound healing have been found to be more frequent in the inflammation and/or proliferation phases and depend on the interactions between different cell types and extracellular matrix, predominantly synthesized by fibroblasts [18].

Wounds may have vascular, traumatic, inflammatory, infectious, or malignant lesions. Acute scarring occurs along a coordinate biochemical cascade; however, a wound may become chronic if the inflammatory and proliferative phases of the cascade suffer some imbalance. Chronic wounds are prevalent and cause substantial morbidity, mortality, and increased health costs [181].

Among the chronic injuries types are venous leg ulcers, common in the elderly and resulting from chronic venous hypertension, characterized by persistent inflammation, hemosiderin deposition, and lipodermatosclerosis [182].

## 7. Pharmacological Interventions

The skin wounds treatment is dynamic and depends of the healing phases evolution. There are numerous curative options on the market that are in the range of simplest coverage, such as hygiene and antisepsis solutions, ointments, gels, growth factors, and even the most complex dressings types called “smart dressings” or “bioactive” [183].

To direct the choosing process of which therapy to use, Das and Baker (2016) [201] emphasized that an accurate lesion assessment is essential, carefully identifying the healing process stage. In addition, the benefits and costs evaluation are some of the aspects to be considered when choosing the treatment type, which should be appropriate to the nature, location, and size of the wound. Although there are a wide variety of dressings, a single type does not meet the requirements to be applied to all cutaneous wounds types.

According to Sibbald et al. (2015) [183], the used therapies for healing can be classified as follows:

- Resources intended to skin protect against mechanical or chemical aggression and infection prevention
- Hygiene and antisepsis products
- Products for chemical, enzymatic, autolytic, or mechanical debridement
- Primary covers (come in direct contact with wound bed) or secondary (they serve to fix primary covers)
- Products for fastening covers and accessories (bands, bandages)
- Topical agents.

Ointments containing debris agents (DNAse, collagenases, fibrinolysins, and papain) are topical options that act selectively promoting a smooth enzymatic digestion on devitalized tissues but present low efficacy in chronic injuries treatment [202]. Dressings with hydrocolloids are also used, which aid in autolytic debridement and stimulate angiogenesis but may cause maceration of adjacent tissue, like calcium alginate, activated charcoal, and hydropolymer adhesive dressing, indicated for exudative wounds and contraindicated for dry wounds [4].

Molecules directly involved in the physiological healing process have also been studied as potential therapeutic targets, since ulcers that are difficult to heal are related to lower expression of these factors. Examples of molecules are PDGF (REGRANEX®) and VEGF, in addition to proteases and degrading agents [32], but the high cost of these therapies makes access to them difficult for the population.

The traditional therapies used in healing have also been strongly practiced, since medicinal plants have historically proven their value as molecules source with therapeutic potential and nowadays still represent an important target for new drugs identification [203]. The medicinal plants scientific evidence on wound healing indicates beneficial effects in different lesions treatment [204–207]. The good manufacturing practices development and regulatory legislation also plays a key role in stimulating the traditional therapies used by clinicians and promote their integration into national health system, since there is widespread acceptance by population.

In this sense, it is important to remember that Brazilian Ministry of Health has stimulated the insertion of complementary care practices in the health system. The implementation of the National Policy of Medicinal Plants and Phytotherapy (PNPMF, in Portuguese) [208] and National Policy on Integrative and Complementary Practices (PNPIC, in Portuguese) [209], which aim to stimulate access to complementary practices and medicinal plants for health care in an effective and safe way, is worth noting.

Another important publication is National Relation of Medicinal Plants of interest to Unified Health System (SUS, in Portuguese) launched in 2009, containing 71 medicinal plants that should be object of research and implementation of the Brazilian public health sectors and services [210]. The Collegiate Board of Directors Resolution (DRC, in Portuguese) number 10, of the year 2010 (a and b), lists 66 medicinal plants with proven actions in human health. Among these, several species are indicated for the cicatrization process, which implies a great advance in the Brazilian public health that begins to value the use of new therapies based on medicinal plants, a practice so widespread around the world and generations.

The chemical compounds present in plants are involved in a variety of steps in healing, ranging from the inflammatory process control to the granulation tissue formation, increase of the lesion contraction, and collagen deposition [211]. Pereira and Bártolo (2016) [212] described a review of some traditional therapies most used in healing of cutaneous injuries, which can be seen in Table 1.

It is noted that the pharmacological effects on healing observed in the plants described in the table may be related to secondary metabolites presence found in these plant materials, since several studies have shown that mainly tannins [213], flavonoids [214], triterpenes [215], and essential oils [216] may be associated with such activity.

In view of the tissue repair response complexity, it is perceived that the treatment with a single factor or cellular component reaches limited effectiveness in the healing of chronic wounds. The challenge lies in the combined therapeutic approaches development or preferably in the products development having more than one biologically active compound, such as a product that stimulates both angiogenesis and matrix deposition, and epithelial migration [5]. So, researchers' attention to factors that delay or accelerate wound healing is important in order to increase the therapeutic arsenal and make wound healing more effective.

## Figures and Tables

**Table 1:** 

Herb	Main constituents	Laboratorial and clinical evidence	References
*Aloe vera*	Soluble sugars, nonstarch polysaccharides, lignin, polysaccharides, glycoproteins, and antiseptic agents	Anti-inflammatory and antimicrobial activities; stimulates cell proliferation, collagen synthesis, and angiogenesis; promotes wound contraction	[184–187]
*Hippophae rhamnoides *(sea buckthorn)	Flavonoids (e.g., quercetin, isorhamnetin), carotenoids (e.g., a-, b-carotene, lycopene), vitamins (C, E, K), tannins, organic acids, triterpenes, glycerides of palmitic, stearic, oleic acids, and amino acids	Antioxidant and anti-inflammatory activities; stimulates the healing process; improves wound contraction and epithelialization; increases the hydroxyproline and protein content in the wound	[188, 189]
*Angelica sinensis*	Essential oils and water-soluble ingredients; ferulic acid is the main active constituent	Stimulates the proliferation of human skin fibroblasts, the secretion of collagen, and the expression of TGF-*β* in vitro	[190]
*Catharanthus roseus (Vinca rosea)*	Contains two major classes of active compounds: alkaloids (e.g., vincamine) and tannins	Antimicrobial activity against *Pseudomonas aeruginosa* and *Staphylococcus aureus*; increases wound strength, epithelialization, and wound contraction	[191]
*Calendula officinalis* (marigold)	Triterpenoids and flavonoids	Anti-inflammatory and antibacterial activities; stimulates the proliferation and migration of fibroblasts in vitro; stimulates the collagen production and angiogenesis	[192–194]
*Sesamum indicum*	Sesamol is the main antioxidant constituent; others include sesamolin and sesaminol	Improves the wound tensile strength, wound contraction, and the hydroxyproline levels in both normal and delayed wound models in rats	[195]
*Morinda citrifolia* (noni)	Acids, alcohols, phenols, esters, anthraquinones, sterols, flavonoids, triterpenoids, saccharides, carotenoids, esters, ketones, lactones, lignans, and nucleosides	Improves the hydroxyproline content and reduces both the wound area and the epithelialization time in excision wounds in rats	[196, 197]
*Camellia sinensis*	Polyphenols, flavonoids, tannins, caffeine, and amino acids	Reduces the healing time and the wound length of incision wounds created in Wistar rats	[198, 199]
*Rosmarinus officinalis* L. (rosemary)	Most bioactive constituents include terpenoids and polyphenols, such as carnosol, carnosic acid, and rosmarinic acid	Reduces the inflammation and improves the wound contraction, reepithelialization, angiogenesis, and collagen deposition on full-thickness wounds in diabetic mice	[200]

## References

[1] Parham P. (2011). *O sistema imune*.

[2] Nicholson L. B. (2016). The imune system. *Essays Biochem*.

[3] Slavich G. M., Irwin M. R. (2014). From stress to inflammation and major depressive disorder: a social signal transduction theory of depression. *Psychological Bulletin*.

[4] Demidova-Rice T. N., Hamblin M. R., Herman I. M. (2012). Acute and impaired wound healing: Pathophysiology and current methods for drug delivery, part 1: normal and chronic wounds: biology, causes, and approaches to care. *Advances in Skin and Wound Care*.

[5] Eming S. A., Martin P., Tomic-Canic M. (2014). Wound repair and regeneration: mechanisms, signaling, and translation. *Science Translational Medicine*.

[6] You H.-J., Han S.-K. (2014). Cell therapy for wound healing. *Journal of Korean Medical Science*.

[7] MacLeod A. S., Rudolph R., Corriden R., Ye I., Garijo O., Havran W. L. (2014). Skin-resident T cells sense ultraviolet radiation-induced injury and contribute to DNA repair. *Journal of Immunology*.

[8] Nussbaum C., Bannenberg S., Keul P. (2015). Sphingosine-1-phosphate receptor 3 promotes leukocyte rolling by mobilizing endothelial P-selectin. *Nature Communications*.

[9] Noda K., Nakao S., Ishida S., Ishibashi T. (2012). Leukocyte adhesion molecules in diabetic retinopathy. *Journal of Ophthalmology*.

[10] Mayadas T. N., Cullere X., Lowell C. A. (2014). The multifaceted functions of neutrophils. *Annual Review of Pathology*.

[11] Kolaczkowska E., Kubes P. (2013). Neutrophil recruitment and function in health and inflammation. *Nature Reviews Immunology*.

[12] Wilgus T. A., Roy S., McDaniel J. C. (2013). Neutrophils and wound repair: positive actions and negative reactions. *Advances in Wound Care*.

[13] De Oliveira S., Rosowski E. E., Huttenlocher A. (2016). Neutrophil migration in infection and wound repair: going forward in reverse. *Nature Reviews Immunology*.

[14] Murray P. J., Wynn T. A. (2011). Protective and pathogenic functions of macrophage subsets. *Nature Reviews Immunology*.

[15] Galván-Peña S., O'Neill L. A. J. (2014). Metabolic reprogramming in macrophage polarization. *Frontiers in Immunology*.

[16] Novak M. L., Koh T. J. (2013). Macrophage phenotypes during tissue repair. *Journal of Leukocyte Biology*.

[17] Mantovani A., Biswas S. K., Galdiero M. R., Sica A., Locati M. (2013). Macrophage plasticity and polarization in tissue repair and remodelling. *The Journal of Pathology*.

[18] Landén N. X., Li D., Ståhle M. (2016). Transition from inflammation to proliferation: a critical step during wound healing. *Cellular and Molecular Life Sciences*.

[19] Pastar I., Stojadinovic O., Yin N. C. (2014). Epithelialization in wound healing: a comprehensive review. *Advances in Wound Care*.

[20] Levinson H. (2013). A paradigm of fibroblast activation and dermal wound contraction to guide the development of therapies for chronic wounds and pathologic scars. *Advances in Wound Care*.

[21] Dipietro L. A. (2013). Angiogenesis and scar formation in healing wounds. *Current Opinion in Rheumatology*.

[22] Ganeshkumar M., Ponrasu T., Krithika R., Iyappan K., Gayathri V. S., Suguna L. (2012). Topical application of *Acalypha indica* accelerates rat cutaneous wound healing by up-regulating the expression of Type I and III collagen. *Journal of Ethnopharmacology*.

[23] Penn J. W., Grobbelaar O. A., Rolfe K. J. (2012). The role of the TGF-*β* family in wound healing, burns and scarring: a review. *International Journal of Burns and Trauma*.

[24] Siviero F. (2013). *Biologia cellular: bases moleculares e metodologia de pesquisa*.

[25] Feng Y., Sanders A. J., Morgan L. D., Harding K. G., Jiang W. G. (2016). Potential roles of suppressor of cytokine signaling in wound healing. *Regenerative Medicine*.

[26] Chen C.-S., Su W.-H., Cheng M.-H. (2010). Nonsteroidal anti-inflammatory drugs for wounds: Pain relief or excessive scar formation?. *Mediators of Inflammation*.

[27] Dinarello C. A. (1984). Interleukin-1 and the pathogenesis of the acute-phase response. *The New England Journal of Medicine*.

[28] Basbaum A. I., Julius D. (2006). Novos alvos contra a dor. *Scientific American Brasil*.

[29] Verri W. A., Cunha T. M., Parada C. A., Poole S., Cunha F. Q., Ferreira S. H. (2006). Hypernociceptive role of cytokines and chemokines: targets for analgesic drug development?. *Pharmacology & Therapeutics*.

[30] Kummer C. L., Coelho T. C. (2002). Antiinflamatórios não esteróides inibidores da ciclooxigenase-2 (COX-2): aspectos atuais. *Revista Brasileira de Anestesiologia*.

[31] Kvaternick V., Pollmeier M., Fischer J., Hanson P. D. (2007). Pharmacokinetics and metabolism of orally administered firocoxib, a novel second generation coxib, in horses. *Journal of Veterinary Pharmacology and Therapeutics*.

[32] Yedgar S., Krimsky M., Cohen Y., Flower R. J. (2007). Treatment of inflammatory diseases by selective eicosanoid inhibition: a double-edged sword?. *Trends in Pharmacological Sciences*.

[33] Gragnani A., Müller B. R., da Silva I. D. C. G., de Noronha S. M. R., Ferreira L. M. (2013). Keratinocyte growth factor, tumor necrosis factor-alpha and interleukin-1 beta gene expression in cultured fibroblasts and keratinocytes from burned patients. *Acta Cirurgica Brasileira*.

[34] Barrientos S., Brem H., Stojadinovic O., Tomic-Canic M. (2014). Clinical application of growth factors and cytokines in wound healing. *Wound Repair and Regeneration*.

[35] Brauchle M., Angermeyer K., Hübner G., Werner S. (1994). Large induction of keratinocyte growth factor expression by serum growth factors and pro-inflammatory cytokines in cultured fibroblasts. *Oncogene*.

[36] Vanezis P. (2016). Book review: the wound healing process: forensic pathological aspects. *Medicine, Science and the Law*.

[37] Kubiczkova L., Sedlarikova L., Hajek R., Sevcikova S. (2012). TGF-*β*—an excellent servant but a bad master. *Journal of Translational Medicine*.

[38] Cury V., Moretti A. I. S., Assis L. (2013). Low level laser therapy increases angiogenesis in a model of ischemic skin flap in rats mediated by VEGF, HIF-1*α* and MMP-2. *Journal of Photochemistry and Photobiology B: Biology*.

[39] Johnson K. E., Wilgus T. A. (2014). Vascular endothelial growth factor and angiogenesis in the regulation of cutaneous wound repair. *Advances in Wound Care*.

[40] Carmeliet P. (2003). Angiogenesis in health and disease. *Nature Medicine*.

[41] Rossiter H., Barresi C., Pammer J. (2004). Loss of vascular endothelial growth factor A activity in murine epidermal keratinocytes delays wound healing and inhibits tumor formation. *Cancer Research*.

[42] Ko H. C., Milthorpe B. K., McFarland C. D. (2007). Engineering thick tissues-the vascularization problem. *European Cells & Materials*.

[43] Kim J., Mirando A. C., Popel A. S., Green J. J. (2016). Gene delivery nanoparticles to modulate angiogenesis. *Advanced Drug Delivery Reviews*.

[44] Detmar M., Brown L. F., Schön M. P. (1998). Increased microvascular density and enhanced leukocyte rolling and adhesion in the skin of VEGF transgenic mice. *Journal of Investigative Dermatology*.

[45] Hong Y.-K., Lange-Asschenfeldt B., Velasco P. (2004). VEGF-A promotes tissue repair-associated lymphatic vessel formation via VEGFR-2 and the *α*1*β*1 and *α*2*β*1 integrins. *FASEB Journal*.

[46] Wulff B. C., Wilgus T. A. (2013). Mast cell activity in the healing wound: more than meets the eye?. *Experimental Dermatology*.

[47] Wilgus T. A., Matthies A. M., Radek K. A. (2005). Novel function for vascular endothelial growth factor receptor-1 on epidermal keratinocytes. *American Journal of Pathology*.

[48] Lucas T., Waisman A., Ranjan R. (2010). Differential roles of macrophages in diverse phases of skin repair. *Journal of Immunology*.

[49] Lauer G., Sollberg S., Cole M. (2000). Expression and proteolysis of vascular endothelial growth factor is increased in chronic wounds. *Journal of Investigative Dermatology*.

[50] Galiano R. D., Tepper O. M., Pelo C. R. (2004). Topical vascular endothelial growth factor accelerates diabetic wound healing through increased angiogenesis and by mobilizing and recruiting bone marrow-derived cells. *The American Journal of Pathology*.

[51] Stallmeyer B., Pfeilschifter J., Frank S. (2001). Systemically and topically supplemented leptin fails to reconstitute a normal angiogenic response during skin repair in diabetic ob/ob mice. *Diabetologia*.

[52] Romano Di Peppe S., Mangoni A., Zambruno G. (2002). Adenovirus-mediated VEGF165 gene transfer enhances wound healing by promoting angiogenesis in CD1 diabetic mice. *Gene Therapy*.

[53] Galeano M., Deodato B., Altavilla D. (2003). Adeno-associated viral vector-mediated human vascular endothelial growth factor gene transfer stimulates angiogenesis and wound healing in the genetically diabetic mouse. *Diabetologia*.

[54] Brem H., Kodra A., Golinko M. S. (2009). Mechanism of sustained release of vascular endothelial growth factor in accelerating experimental diabetic healing. *Journal of Investigative Dermatology*.

[55] Gosain A., DiPietro L. A. (2004). Aging and wound healing. *World Journal of Surgery*.

[56] Campos A. C. L., Groth A. K., Branco A. B. (2008). Assessment and nutritional aspects of wound healing. *Current Opinion in Clinical Nutrition and Metabolic Care*.

[57] Sakamaki K. (2004). Regulation of endothelial cell death and its role in angiogenesis and vascular regression. *Current Neurovascular Research*.

[58] Dimmeler S., Zeiher A. M. (2000). Endothelial cell apoptosis in angiogenesis and vessel regression. *Circulation Research*.

[59] Stoneman V. E. A., Bennett M. R. (2009). Role of Fas/Fas-L in vascular cell apoptosis. *Journal of Cardiovascular Pharmacology*.

[60] Volpert O. V., Zaichuk T., Zhou W. (2002). Inducer-stimulated Fas targets activated endothelium for destruction by anti-angiogenic thrombospondin-1 and pigment epithelium-derived factor. *Nature Medicine*.

[61] Gosain A., Matthies A. M., Dovi J. V., Barbul A., Gamelli R. L., DiPietro L. A. (2006). Exogenous pro-angiogenic stimuli cannot prevent physiologic vessel regression. *Journal of Surgical Research*.

[62] Sorrell J. M., Baber M. A., Caplan A. I. (2008). Human dermal fibroblast subpopulations; Differential interactions with vascular endothelial cells in coculture: nonsoluble factors in the extracellular matrix influence interactions. *Wound Repair and Regeneration*.

[63] Suhr F., Brixius K., Bloch W. (2009). Angiogenic and vascular modulation by extracellular matrix cleavage products. *Current Pharmaceutical Design*.

[64] Miura S., Mitsui K., Heishi T. (2010). Impairment of VEGF-A-stimulated lamellipodial extensions and motility of vascular endothelial cells by chondromodulin-I, a cartilage-derived angiogenesis inhibitor. *Experimental Cell Research*.

[65] Loegl J., Nussbaumer E., Hiden U. (2016). Pigment epithelium-derived factor (PEDF): a novel trophoblast-derived factor limiting feto-placental angiogenesis in late pregnancy. *Angiogenesis*.

[66] Maestroni S., Maestroni A., Ceglia S. (2015). Effect of chromogranin A-derived vasostatin-1 on laser-induced choroidal neovascularization in the mouse. *Acta Ophthalmologica*.

[67] Kamphaus G. D., Colorado P. C., Panka D. J. (2000). Canstatin, a novel matrix-derived inhibitor of angiogenesis and tumor growth. *Journal of Biological Chemistry*.

[68] Hamano Y., Zeisberg M., Sugimoto H. (2003). Physiological levels of tumstatin, a fragment of collagen IV *α*3 chain, are generated by MMP-9 proteolysis and suppress angiogenesis via *α*V*β*3 integrin. *Cancer Cell*.

[69] Nyberg P., Xie L., Sugimoto H. (2008). Characterization of the anti-angiogenic properties of arresten, an *α*1*β*1 integrin-dependent collagen-derived tumor suppressor. *Experimental Cell Research*.

[70] Murphy A. N., Unsworth E. J., Stetler‐Stevenson W. G. (1993). Tissue inhibitor of metalloproteinases‐2 inhibits bFGF‐induced human microvascular endothelial cell proliferation. *Journal of Cellular Physiology*.

[71] Fernandez H. A., Kallenbach K., Seghezzi G. (1999). Inhibition of endothelial cell migration by gene transfer of tissue inhibitor of metalloproteinases-1. *Journal of Surgical Research*.

[72] O'Reilly M. S., Boehm T., Shing Y. (1997). Endostatin: an endogenous inhibitor of angiogenesis and tumor growth. *Cell*.

[73] Pinessi D., Foglieni C., Bugatti A. (2016). PO-15—antiangiogenic small molecule ligands of FGF2 derived from the endogenous inhibitor thrombospondin-1. *Thrombosis Research*.

[74] Duda D. G., Sunamura M., Lozonschi L. (2000). Direct in vitro evidence and in vivo analysis of the antiangiogenesis effects of interleukin 12. *Cancer Research*.

[75] Cai W.-B., Zhang Y., Cheng R. (2012). Dual inhibition of plasminogen kringle 5 on angiogenesis and chemotaxis suppresses tumor metastasis by targeting HIF-1*α* pathway. *PLoS ONE*.

[76] Jonasch E., Haluska F. G. (2001). Interferon in oncological practice: review of interferon biology, clinical applications, and toxicities. *Oncologist*.

[77] Dawson D. W., Volpert O. V., Gillis P. (1999). Pigment epithelium-derived factor: a potent inhibitor of angiogenesis. *Science*.

[78] Ho T.-C., Chen S.-L., Yang Y.-C., Liao C.-L., Cheng H.-C., Tsao Y.-P. (2007). PEDF induces p53-mediated apoptosis through PPAR gamma signaling in human umbilical vein endothelial cells. *Cardiovascular Research*.

[79] Aurora A. B., Biyashev D., Mirochnik Y. (2010). NF-kappaB balances vascular regression and angiogenesis via chromatin remodeling and NFAT displacement. *Blood*.

[80] Slaton J. W., Perrotte P., Inoue K., Dinney C. P. N., Fidler I. J. (1999). Interferon-*α*-mediated down-regulation of angiogenesis-related genes and therapy of bladder cancer are dependent on optimization of biological dose and schedule. *Clinical Cancer Research*.

[81] Ahmed A., Ahmad S., Hewett P. W. (2011). Autocrine activity of soluble Flt-1 controls endothelial cell function and angiogenesis. *Vascular Cell*.

[82] Kim Y.-M., Hwang S., Pyun B.-J. (2002). Endostatin blocks vascular endothelial growth factor-mediated signaling via direct interaction with KDR/Flk-1. *The Journal of Biological Chemistry*.

[83] Huang S., Ullrich S. E., Bar-Eli M. (1999). Regulation of tumor growth and metastasis by interleukin-10: the melanoma experience. *Journal of Interferon and Cytokine Research*.

[84] Claesson-Welsh L., Welsh M., Ito N. (1998). Angiostatin induces endothelial cell apoptosis and activation of focal adhesion kinase independently of the integrin-binding motif RGD. *Proceedings of the National Academy of Sciences of the United States of America*.

[85] Eriksson K., Magnusson P., Dixelius J., Claesson-Welsh L., Cross M. J. (2003). Angiostatin and endostatin inhibit endothelial cell migration in response to FGF and VEGF without interfering with specific intracellular signal transduction pathways. *FEBS Letters*.

[86] Griscelli F., Li H., Bennaceur-Griscelli A. (1998). Angiostatin gene transfer: inhibition of tumor growth in vivo by blockage of endothelial cell proliferation associated with a mitosis arrest. *Proceedings of the National Academy of Sciences of the United States of America*.

[87] Moser T. L., Stack M. S., Asplin I. (1999). Angiostatin binds ATP synthase on the surface of human endothelial cells. *Proceedings of the National Academy of Sciences of the United States of America*.

[88] Hajitou A., Grignet C., Devy L. (2002). The antitumoral effect of endostatin and angiostatin is associated with a down-regulation of vascular endothelial growth factor expression in tumor cells. *The FASEB Journal*.

[89] Lawler P. R., Lawler J. (2012). Molecular basis for the regulation of angiogenesis by thrombospondin-1 and -2. *Cold Spring Harbor Perspectives in Medicine*.

[90] Kyriakides T. R., MacLauchlan S. (2009). The role of thrombospondins in wound healing, ischemia, and the foreign body reaction. *Journal of Cell Communication and Signaling*.

[91] Couper K. N., Blount D. G., Riley E. M. (2008). IL-10: the master regulator of immunity to infection. *Journal of Immunology*.

[92] Wu W.-K., Llewellyn O. P. C., Bates D. O., Nicholson L. B., Dick A. D. (2010). IL-10 regulation of macrophage VEGF production is dependent on macrophage polarisation and hypoxia. *Immunobiology*.

[93] Yamamoto T., Eckes B., Krieg T. (2001). Effect of Interleukin-10 on the gene expression of type I collagen, fibronectin, and decorin in human skin fibroblasts: differential regulation by transforming growth factor-*β* and monocyte chemoattractant protein-1. *Biochemical and Biophysical Research Communications*.

[94] Kieran I., Knock A., Bush J. (2013). Interleukin-10 reduces scar formation in both animal and human cutaneous wounds: Results of two preclinical and phase II randomized control studies. *Wound Repair and Regeneration*.

[95] Wilczynska A., Bushell M. (2015). The complexity of miRNA-mediated repression. *Cell Death and Differentiation*.

[96] Icli B., Nabzdyk C. S., Lujan-Hernandez J. (2016). Regulation of impaired angiogenesis in diabetic dermal wound healing by microRNA-26a. *Journal of Molecular and Cellular Cardiology*.

[97] Banerjee J., Sen C. K. (2015). Microrna and wound healing. *Advances in Experimental Medicine and Biology*.

[98] He L., Hannon G. J. (2004). MicroRNAs: small RNAs with a big role in gene regulation. *Nature Reviews Genetics*.

[99] Shilo S., Roy S., Khanna S., Sen C. K. (2007). MicroRNA in cutaneous wound healing: a new paradigm. *DNA and Cell Biology*.

[100] Gregory R. I., Chendrimada T. P., Shiekhattar R. (2006). MicroRNA biogenesis: isolation and characterization of the microprocessor complex. *Methods in Molecular Biology*.

[101] Park H., Huang X., Lu C., Cairo M. S., Zhou X. (2015). MicroRNA-146a and microRNA-146b regulate human dendritic cell apoptosis and cytokine production by targeting TRAF6 and IRAK1 proteins. *Journal of Biological Chemistry*.

[102] Sheedy F. J., Palsson-Mcdermott E., Hennessy E. J. (2010). Negative regulation of TLR4 via targeting of the proinflammatory tumor suppressor PDCD4 by the microRNA miR-21. *Nature Immunology*.

[103] Yang X., Wang J., Guo S.-L. (2011). miR-21 promotes keratinocyte migration and re-epithelialization during wound healing. *International Journal of Biological Sciences*.

[104] Xu J., Wu W., Zhang L. (2012). The role of MicroRNA-146a in the pathogenesis of the diabetic wound-healing impairment: correction with mesenchymal stem cell treatment. *Diabetes*.

[105] Fahs F., Bi X., Yu F.-S., Zhou L., Mi Q.-S. (2015). New insights into microRNAs in skin wound healing. *IUBMB Life*.

[106] Wang S., Aurora A. B., Johnson B. A. (2008). The endothelial-specific microRNA miR-126 governs vascular integrity and angiogenesis. *Developmental Cell*.

[107] Bonauer A., Carmona G., Iwasaki M. (2009). MicroRNA-92a controls angiogenesis and functional recovery of ischemic tissues in mice. *Science*.

[108] Fasanaro P., D'Alessandra Y., Di Stefano V. (2008). MicroRNA-210 modulates endothelial cell response to hypoxia and inhibits the receptor tyrosine kinase ligand ephrin-A3. *Journal of Biological Chemistry*.

[109] Chen Y., Gorski D. H. (2008). Regulation of angiogenesis through a microRNA (miR-130a) that down-regulates antiangiogenic homeobox genes *GAX* and *HOXA5*. *Blood*.

[110] Zhou Q., Gallagher R., Ufret-Vincenty R., Li X., Olson E. N., Wang S. (2011). Regulation of angiogenesis and choroidal neovascularization by members of microRNA-23~27~24 clusters. *Proceedings of the National Academy of Sciences of the United States of America*.

[111] Suárez Y., Fernández-Hernando C., Pober J. S., Sessa W. C. (2007). Dicer dependent microRNAs regulate gene expression and functions in human endothelial cells. *Circulation Research*.

[112] Menghini R., Casagrande V., Cardellini M. (2009). MicroRNA 217 modulates endothelial cell senescence via silent information regulator 1. *Circulation*.

[113] Sundaram G. M., Common J. E. A., Gopal F. E. (2013). ‘See-saw’ expression of microRNA-198 and FSTL1 from a single transcript in wound healing. *Nature*.

[114] Viticchiè G., Lena A. M., Cianfarani F. (2012). MicroRNA-203 contributes to skin re-epithelialization. *Cell Death & Disease*.

[115] Bertero T., Gastaldi C., Bourget-Ponzio I. (2011). miR-483-3p controls proliferation in wounded epithelial cells. *The FASEB Journal*.

[116] Ciechomska M., O'Reilly S., Suwara M., Bogunia-Kubik K., Van Laar J. M. (2014). MiR-29a reduces TIMP-1 production by dermal fibroblasts via targeting TGF-*β* activated kinase 1 binding protein 1, implications for systemic sclerosis. *PLoS ONE*.

[117] Wang B., Herman-Edelstein M., Koh P. (2010). E-cadherin expression is regulated by miR-192/215 by a mechanism that is independent of the profibrotic effects of transforming growth factor-*β*. *Diabetes*.

[118] Dey B. K., Gagan J., Yan Z., Dutta A. (2012). miR-26a is required for skeletal muscle differentiation and regeneration in mice. *Genes and Development*.

[119] Icli B., Wara A. K. M., Moslehi J. (2013). MicroRNA-26a regulates pathological and physiological angiogenesis by targeting BMP/SMAD1 signaling. *Circulation Research*.

[120] Huebener P., Schwabe R. F. (2013). Regulation of wound healing and organ fibrosis by toll-like receptors. *Biochimica et Biophysica Acta—Molecular Basis of Disease*.

[121] Lin Q., Li M., Fang D., Fang J., Su S. B. (2011). The essential roles of Toll-like receptor signaling pathways in sterile inflammatory diseases. *International Immunopharmacology*.

[122] Kluwe J., Mencin A., Schwabe R. F. (2009). Toll-like receptors, wound healing, and carcinogenesis. *Journal of Molecular Medicine*.

[123] Voulgarelis M., Ioannou S. (2010). Toll-like receptors, tissue injury, and tumourigenesis. *Mediators of Inflammation*.

[124] Lotze M. T., Zeh H. J., Rubartelli A. (2007). The grateful dead: damage-associated molecular pattern molecules and reduction/oxidation regulate immunity. *Immunological Reviews*.

[125] Beutler B. (2007). Neo-ligands for innate immune receptors and the etiology of sterile inflammatory disease. *Immunological Reviews*.

[126] Lin Q., Wang L., Lin Y. (2012). Toll-like receptor 3 ligand polyinosinic: polycytidylic acid promotes wound healing in human and murine skin. *Journal of Investigative Dermatology*.

[127] Lin Q., Fang D., Fang J. (2011). Impaired wound healing with defective expression of chemokines and recruitment of myeloid cells in TLR3-deficient mice. *Journal of Immunology*.

[128] Gregorio J., Meller S., Conrad C. (2010). Plasmacytoid dendritic cells sense skin injury and promote wound healing through type i interferons. *Journal of Experimental Medicine*.

[129] Sato T., Yamamoto M., Shimosato T., Klinman D. M. (2010). Accelerated wound healing mediated by activation of Toll-like receptor 9. *Wound Repair and Regeneration*.

[130] Yamamoto M., Sato T., Beren J., Verthelyi D., Klinman D. M. (2011). The acceleration of wound healing in primates by the local administration of immunostimulatory CpG oligonucleotides. *Biomaterials*.

[131] Macedo L., Pinhal-Enfield G., Alshits V., Elson G., Cronstein B. N., Leibovich S. J. (2007). Wound healing is impaired in MyD88-deficient mice: a role for MyD88 in the regulation of wound healing by adenosine A2A receptors. *American Journal of Pathology*.

[132] Chen L., Guo S., Ranzer M. J., Dipietro L. A. (2013). Toll-like receptor 4 has an essential role in early skin wound healing. *Journal of Investigative Dermatology*.

[133] Suga H., Sugaya M., Fujita H. (2014). TLR4, rather than TLR2, regulates wound healing through TGF-*β* and CCL5 expression. *Journal of Dermatological Science*.

[134] Portou M., Baker D., Abraham D., Tsui J. (2015). The innate immune system, toll-like receptors and dermal wound healing: a review. *Vascular Pharmacology*.

[135] Dasu M. R., Rivkah Isseroff R. (2012). Toll-like receptors in wound healing: location, accessibility, and timing. *Journal of Investigative Dermatology*.

[136] Anderson K., Hamm R. L. (2012). Factors that impair wound healing. *Journal of the American College of Clinical Wound Specialists*.

[137] Castilla D. M., Liu Z., Velazquez O. C. (2012). Oxygen: implications for wound healing. *Advances in Wound Care*.

[138] Yip W. L. (2015). Influence of oxygen on wound healing. *International Wound Journal*.

[139] Zhang Z., Cao G., Sha L., Wang D., Liu M. (2015). The efficacy of sodium aescinate on cutaneous wound healing in diabetic rats. *Inflammation*.

[140] Gould L., Abadir P., Brem H. (2015). Chronic wound repair and healing in older adults: current status and future research. *Journal of the American Geriatrics Society*.

[141] Gainza G., Villullas S., Pedraz J. L., Hernandez R. M., Igartua M. (2015). Advances in drug delivery systems (DDSs) to release growth factors for wound healing and skin regeneration. *Nanomedicine*.

[142] Zhou T., Yang Z., Chen Y. (2016). Estrogen accelerates cutaneous wound healing by promoting proliferation of epidermal keratinocytes via Erk/Akt signaling pathway. *Cellular Physiology and Biochemistry*.

[143] Son E. D., Lee J. Y., Lee S. (2005). Topical application of 17*β*-estradiol increases extracellular matrix protein synthesis by stimulating TGF-*β* signaling in aged human skin in vivo. *Journal of Investigative Dermatology*.

[144] Thornton K. R., Foran A. J., Long A. D. (2013). Properties and modeling of GWAS when complex disease risk is due to non-complementing, deleterious mutations in genes of large effect. *PLoS Genetics*.

[145] Hardman M. J., Ashcroft G. S. (2008). Estrogen, not intrinsic aging, is the major regulator of delayed human wound healing in the elderly. *Genome Biology*.

[146] Emmerson E., Hardman M. J. (2012). The role of estrogen deficiency in skin ageing and wound healing. *Biogerontology*.

[147] Boyapati L., Wang H.-L. (2007). The role of stress in periodontal disease and wound healing. *Periodontology 2000*.

[148] Romana-Souza B., Assis de Brito T. L., Pereira G. R., Monte-Alto-Costa A. (2014). Gonadal hormones differently modulate cutaneous wound healing of chronically stressed mice. *Brain, Behavior, and Immunity*.

[149] Gajendrareddy P. K., Engeland C. G., Junges R., Horan M. P., Rojas I. G., Marucha P. T. (2013). MMP-8 overexpression and persistence of neutrophils relate to stress-impaired healing and poor collagen architecture in mice. *Brain, Behavior, and Immunity*.

[150] Tymen S. D., Rojas I. G., Zhou X., Fang Z. J., Zhao Y., Marucha P. T. (2013). Restraint stress alters neutrophil and macrophage phenotypes during wound healing. *Brain, Behavior, and Immunity*.

[151] Boulton A. J. M. (2013). The pathway to foot ulceration in diabetes. *Medical Clinics of North America*.

[152] Kolluru G. K., Bir S. C., Kevil C. G. (2012). Endothelial dysfunction and diabetes: effects on angiogenesis, vascular remodeling, and wound healing. *International Journal of Vascular Medicine*.

[153] Baltzis D., Eleftheriadou I., Veves A. (2014). Pathogenesis and treatment of impaired wound healing in diabetes mellitus: new insights. *Advances in Therapy*.

[154] Luevano-Contreras C., Chapman-Novakofski K. (2010). Dietary advanced glycation end products and aging. *Nutrients*.

[155] Kellow N. J., Coughlan M. T. (2015). Effect of diet-derived advanced glycation end products on inflammation. *Nutrition Reviews*.

[156] Pradhan L., Nabzdyk C., Andersen N. D., LoGerfo F. W., Veves A. (2009). Inflammation and neuropeptides: the connection in diabetic wound healing. *Expert Reviews in Molecular Medicine*.

[157] Blakytny R., Jude E. (2006). The molecular biology of chronic wounds and delayed healing in diabetes. *Diabetic Medicine*.

[158] Ahmed N. (2005). Advanced glycation endproducts—role in pathology of diabetic complications. *Diabetes Research and Clinical Practice*.

[159] Khanna S., Biswas S., Shang Y. (2010). Macrophage dysfunction impairs resolution of inflammation in the wounds of diabetic mice. *PLoS ONE*.

[160] Acosta J. B., Garcia Del Barco D., Cibrian Vera D. (2008). The pro-inflammatory environment in recalcitrant diabetic foot wounds. *International Wound Journal*.

[161] Dinh T., Tecilazich F., Kafanas A. (2012). Mechanisms involved in the development and healing of diabetic foot ulceration. *Diabetes*.

[162] Ochoa O., Torres F. M., Shireman P. K. (2007). Chemokines and diabetic wound healing. *Vascular*.

[163] Liu Z.-J., Velazquez O. C. (2008). Hyperoxia, endothelial progenitor cell mobilization, and diabetic wound healing. *Antioxidants and Redox Signaling*.

[164] Peichev M., Naiyer A. J., Pereira D. (2000). Expression of VEGFR-2 and AC133 by circulating human CD34^+^ cells identifies a population of functional endothelial precursors. *Blood*.

[165] Kamihata H., Matsubara H., Nishiue T. (2001). Implantation of bone marrow mononuclear cells into ischemic myocardium enhances collateral perfusion and regional function via side supply of angioblasts, angiogenic ligands, and cytokines. *Circulation*.

[166] Urbich C., Aicher A., Heeschen C. (2005). Soluble factors released by endothelial progenitor cells promote migration of endothelial cells and cardiac resident progenitor cells. *Journal of Molecular and Cellular Cardiology*.

[167] Bruhn-Olszewska B., Korzon-Burakowska A., Gabig-Cimińska M., Olszewski P., Wegrzyn A., Jakóbkiewicz-Banecka J. (2012). Molecular factors involved in the development of diabetic foot syndrome. *Acta Biochimica Polonica*.

[168] Hu S. C.-S., Lan C.-C. E. (2016). High-glucose environment disturbs the physiologic functions of keratinocytes: focusing on diabetic wound healing. *Journal of Dermatological Science*.

[169] Falanga V. (2005). Wound healing and its impairment in the diabetic foot. *The Lancet*.

[170] Kasuya A., Tokura Y. (2014). Attempts to accelerate wound healing. *Journal of Dermatological Science*.

[171] Huang H., Cui W., Qiu W. (2015). Impaired wound healing results from the dysfunction of the Akt/mTOR pathway in diabetic rats. *Journal of Dermatological Science*.

[172] Wang A. S., Armstrong E. J., Armstrong A. W. (2013). Corticosteroids and wound healing: clinical considerations in the perioperative period. *American Journal of Surgery*.

[173] Batlouni M. (2010). Nonsteroidal anti-inflammatory drugs: cardiovascular, cerebrovascular and renal effects. *Arquivos Brasileiros de Cardiologia*.

[174] Erinjeri J. P., Fong A. J., Kemeny N. E., Brown K. T., Getrajdman G. I., Solomon S. B. (2011). Timing of administration of bevacizumab chemotherapy affects wound healing after chest wall port placement. *Cancer*.

[175] Ranzer M. J., Chen L., DiPietro L. A. (2011). Fibroblast function and wound breaking strength is impaired by acute ethanol intoxication. *Alcoholism: Clinical and Experimental Research*.

[176] Sørensen L. T. (2012). Wound healing and infection in surgery: the pathophysiological impact of smoking, smoking cessation, and nicotine replacement therapy: a systematic review. *Annals of Surgery*.

[177] Quain A. M., Khardori N. M. (2015). Nutrition in wound care management: a comprehensive overview. *Wounds*.

[178] Wagner I. J., Szpalski C., Allen R. J. (2012). Obesity impairs wound closure through a vasculogenic mechanism. *Wound Repair and Regeneration*.

[179] Yazdanpanah L., Nasiri M., Adarvishi S. (2015). Literature review on the management of diabetic foot ulcer. *World Journal of Diabetes*.

[180] Pence B. D., Woods J. A. (2014). Exercise, obesity, and cutaneous wound healing: evidence from rodent and human studies. *Advances in Wound Care*.

[181] Paul J. (2013). Characteristics of chronic wounds that itch. *Advances in Skin and Wound Care*.

[182] Dias T. Y. A. F., Costa I. K. F., Melo M. M., de Oliveira Torres S. M. D. S. G. S., Maia M. C. E., de Vasconcelos Torres G. (2014). Quality of life assessment of patients with and without venous ulcer. *Revista Latino-Americana de Enfermagem*.

[183] Sibbald R. G., Elliott J. A., Ayello E. A., Somayaji R. (2015). Optimizing the moisture management tightrope with wound bed preparation 2015©. *Advances in Skin and Wound Care*.

[184] Inpanya P., Faikrua A., Ounaroon A., Sittichokechaiwut A., Viyoch J. (2012). Effects of the blended fibroin/aloe gel film on wound healing in streptozotocin-induced diabetic rats. *Biomedical Materials*.

[185] Tarameshloo M., Norouzian M., Zarein-Dolab S., Dadpay M., Mohsenifar J., Gazor R. (2012). Aloe vera gel and thyroid hormone cream may improve wound healing in Wistar rats. *Anatomy & Cell Biology*.

[186] Atiba A., Nishimura M., Kakinuma S. (2011). Aloe vera oral administration accelerates acute radiation-delayed wound healing by stimulating transforming growth factor-*β* and fibroblast growth factor production. *American Journal of Surgery*.

[187] Atiba A., Ueno H., Uzuka Y. (2011). The effect of aloe vera oral administration on cutaneous wound healing in type 2 diabetic rats. *Journal of Veterinary Medical Science*.

[188] Upadhyay N. K., Kumar R., Mandotra S. K. (2009). Safety and healing efficacy of Sea buckthorn (*Hippophae rhamnoides* L.) seed oil on burn wounds in rats. *Food and Chemical Toxicology*.

[189] Gupta A., Kumar R., Pal K., Singh V., Banerjee P. K., Sawhney R. C. (2006). Influence of sea buckthorn (*Hippophae rhamnoides* L.) flavone on dermal wound healing in rats. *Molecular and Cellular Biochemistry*.

[190] Hsiao C.-Y., Hung C.-Y., Tsai T.-H., Chak K.-F. (2012). A study of the wound healing mechanism of a traditional chinese medicine, *Angelica sinensis*, using a proteomic approach. *Evidence-Based Complementary and Alternative Medicine*.

[191] Nayak B. S., Pinto Pereira L. M. (2006). *Catharanthus roseus* flower extract has wound-healing activity in Sprague Dawley rats. *BMC Complementary and Alternative Medicine*.

[192] Naeini A. T., Miri R., Shafiei N., Tabandeh M. R., Oryan A., Nazifi S. (2012). Effects of topical application of *Calendula officinalis* gel on collagen and hydroxyproline content of skin in rats. *Comparative Clinical Pathology*.

[193] Parente L. M. L., Lino Júnior R. D. S., Tresvenzol L. M. F., Vinaud M. C., De Paula J. R., Paulo N. M. (2012). Wound healing and anti-inflammatory effect in animal models of *Calendula officinalis* L. growing in Brazil. *Evidence-Based Complementary and Alternative Medicine*.

[194] Fronza M., Heinzmann B., Hamburger M., Laufer S., Merfort I. (2009). Determination of the wound healing effect of *Calendula* extracts using the scratch assay with 3T3 fibroblasts. *Journal of Ethnopharmacology*.

[195] Shenoy R. R., Sudheendra A. T., Nayak P. G., Paul P., Kutty N. G., Rao C. M. (2011). Normal and delayed wound healing is improved by sesamol, an active constituent of *Sesamum indicum* (L.) in albino rats. *Journal of Ethnopharmacology*.

[196] Singh D. R. (2012). *Morinda citrifolia* L. (Noni): A review of the scientific validation for its nutritional and therapeutic properties. *Journal of Diabetes and Endocrinology*.

[197] Nayak B. S., Sandiford S., Maxwell A. (2009). Evaluation of the wound-healing activity of ethanolic extract of *Morinda citrifolia* L. leaf. *Evidence-Based Complementary and Alternative Medicine*.

[198] Sharangi A. B. (2009). Medicinal and therapeutic potentialities of tea (*Camellia sinensis* L.)—a review. *Food Research International*.

[199] Asadi S. Y., Parsaei P., Karimi M. (2013). Effect of green tea (*Camellia sinensis*) extract on healing process of surgical wounds in rat. *International Journal of Surgery*.

[200] Abu-Al-Basal M. A. (2010). Healing potential of *Rosmarinus officinalis* L. on full-thickness excision cutaneous wounds in alloxan-induced-diabetic BALB/c mice. *Journal of Ethnopharmacology*.

[201] Das S., Baker A. B. (2016). Biomaterials and nanotherapeutics for enhancing skin wound healing. *Frontiers in Bioengineering and Biotechnology*.

[202] Mendonça R. J., Coutinho-Netto J. (2009). Cellular aspects of wound healing. *Anais Brasileiros de Dermatologia*.

[203] Atanasov A. G., Waltenberger B., Pferschy-Wenzig E. M. (2015). Discovery and resupply of pharmacologically active plant-derived natural products: a review. *Biotechnology Advances*.

[204] Singhal A., Gupta H., Bhati V. (2011). Wound healing activity of *Argyreia nervosa* leaves extract. *International Journal of Applied and Basic Medical Research*.

[205] da Silva Junior I. F., Balogun S. O., de Oliveira R. G., Damazo A. S., Martins D. T. D. O. (2016). *Piper umbellatum* L.: a medicinal plant with gastric-ulcer protective and ulcer healing effects in experimental rodent models. *Journal of Ethnopharmacology*.

[206] Akbik D., Ghadiri M., Chrzanowski W., Rohanizadeh R. (2014). Curcumin as a wound healing agent. *Life Sciences*.

[207] Estevão L. R. M., de Medeiros J. P., Baratella-Evêncio L., Simões R. S., Mendonça F. D. S., Evêncio-Neto J. (2013). Effects of the topical administration of copaiba oil ointment (*Copaifera langsdorffii*) in skin flaps viability of rats. *Acta Cirurgica Brasileira*.

[208] Brasil Ministério da Saúde. Secretaria de Ciência, Tecnologia e Insumos Estratégicos. Departamento de Assistência Farmacêutica. Política nacional de plantas medicinais e fitoterápicos. Brasília: Ministério da Saúde.

[209] Brasil Ministério da Saúde. Secretaria de Atenção à Saúde. Departamento de Atenção Básica. Política Nacional de Práticas Integrativas e Complementares no SUS - PNPIC-SUS/ Ministério da Saúde, Secretaria de Atenção à Saúde, Departamento de Atenção Básica. - Brasília: Ministério da Saúde.

[210] Brasil Ministério da Saúde. Secretaria de Ciência, Tecnologia e Insumos Estratégicos. Departamento de Assistência Farmacêutica e Insumos Estratégicos. Programa Nacional de Plantas Medicinais e Fitoterápicos/ Ministério da Saúde, Secretaria de Ciência, Tecnologia e Insumos Estratégicos, Departamento de Assistência Farmacêutica e Insumos Estratégicos. – Brasília: Ministério da Saúde.

[211] Sivamani R. K., Ma B. R., Wehrli L. N., Maverakis E. (2012). Phytochemicals and naturally derived substances for wound healing. *Advances in Wound Care*.

[212] Pereira R. F., Bártolo P. J. (2016). Traditional therapies for skin wound healing. *Advances in Wound Care*.

[213] Li K., Diao Y., Zhang H. (2011). Tannin extracts from immature fruits of *Terminalia chebula* Fructus Retz. promote cutaneous wound healing in rats. *BMC Complementary and Alternative Medicine*.

[214] Fujita K., Kuge K., Ozawa N. (2015). Cinnamtannin B-1 promotes migration of mesenchymal stem cells and accelerates wound healing in mice. *PLoS ONE*.

[215] Mori H.-M., Kawanami H., Kawahata H., Aoki M. (2016). Wound healing potential of lavender oil by acceleration of granulation and wound contraction through induction of TGF-*β* in a rat model. *BMC Complementary and Alternative Medicine*.

[216] Wu Y.-S., Chen S.-N. (2016). Extracted triterpenes from Antrodia cinnamomea reduce the inflammation to promote the wound healing via the STZ inducing hyperglycemia-diabetes mice model. *Frontiers in Pharmacology*.

